# Supplementary material for: A potent HNF4α agonist reveals that HNF4α controls genes important in inflammatory bowel disease and Paneth cells
Source: PLoS One. 2022 Apr 6;17(4):e0266066. doi: 10.1371/journal.pone.0266066 (PMC8985954; doi:10.1371/journal.pone.0266066)
Supplement: Attachment — Submitted filename: Response to Reviewers.docx [file pone.0266066.s001.docx]

July 7, 2021

Editor

PLoS One

To the Editor,

Please accept for consideration for publication as a research article in PLoS One this revised manuscript (PONE-D-21-30275), titled “A potent HNF4α agonist reveals that HNF4α controls genes important in inflammatory bowel disease and Paneth cells.” Our response to the comments of the reviewers are below.

Thank you for considering our manuscript.

Editor’s Comments

When submitting your revision, we need you to address these additional requirements.

**1.** Please ensure that your manuscript meets PLOS ONE's style requirements, including those for file naming. The PLOS ONE style templates can be found at

https://journals.plos.org/plosone/s/file?id=wjVg/PLOSOne_formatting_sample_main_body.pdf and  https://journals.plos.org/plosone/s/file?id=ba62/PLOSOne_formatting_sample_title_authors_affiliations.pdf

**RESPONSE**: We have edited the manuscript to comply with the style requirements.

**2.** To comply with PLOS ONE submissions requirements, in your Methods section, please provide additional information on the animal research and ensure you have included details on (1) number of animals used in the study, and (2) basic housing.

**RESPONSE:**: Information on the number of mice in each experiment is included and we have added additional information on housing to the Methods.

**3.** Thank you for stating the following in the Acknowledgments Section of your manuscript: 
[We thank the following SBP Core Facilities: Imaging, Animal, Genomics, Conrad Prebys Center for Chemical Genomics, Histology. Sources of Support:  This work was funded by the Sanford Children’s Health Research Center and Brightseed, Inc. FL holds equity in Brightseed.]

We note that you have provided funding information that is not currently declared in your Funding Statement. However, funding information should not appear in the Acknowledgments section or other areas of your manuscript. We will only publish funding information present in the Funding Statement section of the online submission form. 
Please remove any funding-related text from the manuscript and let us know how you would like to update your Funding Statement. Currently, your Funding Statement reads as follows: 
 [This work was funded by the Sanford Children’s Health Research Center and Brightseed, Inc. The funders had no role in study design, data collection and analysis, decision to publish, or preparation of the manuscript.]

Please include your amended statements within your cover letter; we will change the online submission form on your behalf.

**RESPONSE:** As requested, the funding information has been deleted from the Acknowledgments. The Funding Statement is fine as is: “This work was funded by the Sanford Children’s Health Research Center and Brightseed, Inc. The funders had no role in study design, data collection and analysis, decision to publish, or preparation of the manuscript.”

**4.** Thank you for stating the following in the Competing Interests section: 
[I have read the journal's policy and the authors of this manuscript have the following competing interests: FL holds equity in Brightseed.]

Please confirm that this does not alter your adherence to all PLOS ONE policies on sharing data and materials, by including the following statement: "This does not alter our adherence to  PLOS ONE policies on sharing data and materials.” (as detailed online in our guide for authors http://journals.plos.org/plosone/s/competing-interests).  If there are restrictions on sharing of data and/or materials, please state these. Please note that we cannot proceed with consideration of your article until this information has been declared.

Please include your updated Competing Interests statement in your cover letter; we will change the online submission form on your behalf.

**RESPONSE:** The cover letter has been modified as requested.

**Reviewer #1:** In this interesting study, the authors have investigated the therapeutic effect of the potent HNF4α agonist, N-trans caffeoyltyramine (NCT) on gene expression in the intestine. This study is based on previous findings that demonstrated the therapeutic potential of the novel HNF4α activator, NCT in the liver by reversing severe NAFLD symptoms (hepatic steatosis) in diet-induced obese mice. For the current study, vehicle (DMSO) or NCT was administered to high fat diet mice for 14 days (2 weeks) followed by RNAseq, RT-PCR, and Immunofluorescence analysis. The results showed that NCT upregulated genes in the small intestine that play important role in IBD and that are shown to be downregulated in IBD. Moreover, NCT also reversed the decrease in lysozyme expression (Paneth cell marker) in the small intestine in high fat diet fed mice. Impaired Paneth cell function and intestinal barrier integrity have shown to be the hallmarks of IBD. Therefore, these data suggest that HNF4α could be a therapeutic target for IBD and that NCT could act as a candidate therapeutic in treating IBD. The manuscript for the most part is easily understandable, straight-forward and the results are clearly presented. The abstract adequately summarizes the data and is concise. The strength of the manuscript includes the therapeutic relevance of the study with respect to the important role of HNF4α agonist, NCT in the upregulation of genes that are decreased in IBD while the weaknesses relate to the lack of utilizing an established mouse model of intestinal disease (colitis) to examine the beneficial effect of NCT.


**CRITIQUE 1**. Since most of the IBD associated genes upregulated by NCT are independent of HFD (high fat diet) effects (Fig. 3E), then it must be assumed that NCT will also show a similar phenomenon in mice fed with normal chow diet. The authors should elaborate and discuss more on this aspect.
**RESPONSE:** The reviewer is correct that most of the IBD-associated genes were not affected by HFD. We agree that this point is worthy of greater emphasis and so have modified the manuscript to make this point more clearly (Lines 210-212, 255-256).

**CRITIQUE 2.** Although, the immunofluorescence (IF) images are convincing (Fig. 1A), it will be important to corroborate IF results with Western blot utilizing small intestinal tissue lysates and specific HNF4α antibody.

**RESPONSE:** To address the reviewer’s request for additional data on HNF4a expression, we performed RT-PCR analysis for HNF4a mRNA, finding that there was a significant increase induced by NCT. That has been added as Figure 1C.

**CRITIQUE 3.** In addition to the lysozyme expression (Paneth cell marker), the authors should also show the altered expression of certain Paneth cell defensins in HFD mice with or without NCT by qRT-PCR.
**RESPONSE.** To address the concern of the reviewer, we have added additional data (Figure 4C, D).

**CRITIQUE 4.** It is not clear which region of the small intestine (jejunum vs ileum) was used for the studies?

**RESPONSE.** Ten cm of intestine proximal to the duodenum, and so consisting primarily of jejunum, was removed. This information has been added to the Materials and Methods- section on Mice.

**Reviewer #2:** Study by Seung- Hee Lee et al- Entitled “A potent HNF4α agonist reveals that HNF4α controls genes important in inflammatory bowel disease and Paneth cells” has interesting findings. Although, there are some concerns that need to be addressed to strengthen the manuscript.

**Major concerns:**

**CRITIQUE 1**. In this study, the authors have showed that the decreased expression of the IBD associated gene and Paneth cell markers by HFD and their reversal by NCT. However, the data does not suggest that the effects are directly due to HNF4A, so it will be important to examine the activation of HNF4A by NCT.

**RESPONSE.** The issue of whether NCT interacts directly with HNF4a is an important question. Our previous work with NCT examined the issue of direct interaction and we have modified the manuscript to make this important point requested by the reviewer (Lines (79-81).

**CRITIQUE 2**. ChIP assays or other studies should be provided to prove that NCT increases the binding of HNF4A to the promoter of its target gene.

**RESPONSE.** The reviewer requests ChIP assays to determine whether the effects of NCT on downstream genes are direct or indirect. Fortunately, a comprehensive ChIP-Seq analysis of the role of HNF4 in the intestine was published recently: Chen L, et al. The nuclear receptor HNF4 drives a brush border gene program conserved across murine intestine, kidney, and embryonic yolk sac. Nat Commun. 2021 May 17;12(1):2886. doi: 10.1038/s41467-021-22761-5. PMID: 34001900; PMCID: PMC8129143. They did not find that the Paneth cell genes that we studied to be direct targets of HNF4a, but a few of the genes associated with IBD were identified as direct targets of HNF4a. The manuscript has been modified to discuss more extensively the results from this paper along with additional discussion of the important issue identified by the reviewer about direct versus indirect effects of HNF4a (Lines 266-270).

**CRITIQUE 3.** Authors should also examine or provide detail that NCT is a specific agonist only to HNF4A, and it is not producing effects indirectly.
**RESPONSE.** As pointed out by the reviewer, the specificity of small molecule compounds is always a concern. We have examined this issue in our previous paper on NCT using siRNA to HNF4a, finding that it ablated the effect of NCT in cultured cells (Lines (79-81).

**Minor concerns:**

1. Authors should rephrase the abstract for better grammar and flow.

**RESPONSE.** The abstract has been edited as requested.

2. Detail of RT-PCR primers and sting analysis should be provided in the methods and materials section.

**RESPONSE.** This information has been added as Table 1.

3. Authors should include the previous study done regarding regulation of stem cells in HNF4A KO mice

**RESPONSE.** We have added a citation to a recent paper on the role of HNF4a in intestinal stem cell renewal (Chen L, et al. HNF4 Regulates Fatty Acid Oxidation and Is Required for Renewal of Intestinal Stem Cells in Mice. Gastroenterology. 2020 Mar;158(4):985-999.e9. doi: 10.1053/j.gastro.2019.11.031. Epub 2019 Nov 22. PMID: 31759926; PMCID: PMC7062567) and hope that this is the citation requested by the reviewer.

4. Authors should also include a column in table 2 for associated diseases same as table 1.

**RESPONSE.** We did not include a column for associated diseases in table 2, since that table included only genes expressed in Paneth cells and the point of the table is to emphasize that many genes expressed in Paneth cells were affected by NCT. To the best of our knowledge, the only disease that has been well studied with regard to Paneth cells is IBD, an association that is discussed at length in the manuscript.

Sincerely,

Fred Levine

SBP Medical Discovery Institute
